# Broad Clade 2 Cross-Reactive Immunity Induced by an Adjuvanted Clade 1 rH5N1 Pandemic Influenza Vaccine

**DOI:** 10.1371/journal.pone.0001665

**Published:** 2008-02-27

**Authors:** Isabel Leroux-Roels, Roger Bernhard, Pascal Gérard, Mamadou Dramé, Emmanuel Hanon, Geert Leroux-Roels

**Affiliations:** 1 Centre for Vaccinology, Ghent University and Hospital, Ghent, Belgium; 2 GlaxoSmithKline Biologicals, Dresden, Germany; 3 GlaxoSmithKline Biologicals, Rixensart, Belgium; Federal University of Sao Paulo, Brazil

## Abstract

**Background:**

The availability of H5N1 vaccines that can elicit a broad cross-protective immunity against different currently circulating clade 2 H5N1 viruses is a pre-requisite for the development of a successful pre-pandemic vaccination strategy. In this regard, it has recently been shown that adjuvantation of a recombinant clade 1 H5N1 inactivated split-virion vaccine with an oil-in-water emulsion-based adjuvant system also promoted cross-immunity against a recent clade 2 H5N1 isolate (A/Indonesia/5/2005, subclade 2.1). Here we further analyse the cross-protective potential of the vaccine against two other recent clade 2 isolates (A/turkey/Turkey/1/2005 and A/Anhui/1/2005 which are, as defined by WHO, representatives of subclades 2.2 and 2.3 respectively).

**Methods and Findings:**

Two doses of the recombinant A/Vietnam/1194/2004 (H5N1, clade 1) vaccine were administered 21 days apart to volunteers aged 18–60 years. We studied the cross-clade immunogenicity of the lowest antigen dose (3.8 µg haemagglutinin) given with (N = 20) or without adjuvant (N = 20). Immune responses were assessed at 21 days following the first and second vaccine doses and at 6 months following first vaccination. Vaccination with two doses of 3.8 µg of the adjuvanted vaccine induced four-fold neutralising seroconversion rates in 85% of subjects against A/turkey/Turkey/1/2005 (subclade 2.2) and 75% of subjects against A/Anhui/1/2005 (subclade 2.3) recombinant strains. There was no response induced against these strains in the non-adjuvanted group. At 6 months following vaccination, 70% and 60% of subjects retained neutralising antibodies against the recombinant subclade 2.2 and 2.3 strains, respectively and 40% of subjects retained antibodies against the recombinant subclade 2.1 A/Indonesia/5/2005 strain.

**Conclusions:**

In addition to antigen dose-sparing, adjuvantation of inactivated split H5N1 vaccine promotes broad and persistent cross-clade immunity which is a pre-requisite for a pre-pandemic vaccine.

**Trial Registration:**

ClinicalTrials.gov NCT00309634

## Introduction

It is widely feared that the ongoing global spread of the highly pathogenic avian H5N1 influenza virus in wild birds and poultry will trigger the next human influenza pandemic [1]–[7]. The H5N1 virus currently fulfils two of the three pre-requisites for a global influenza pandemic to occur [1]. First H5 is a new haemagglutinin (HA) subtype to which virtually the entire human population lacks immunity. Second the virus can replicate in humans and cause serious illness and death. The first human disease caused by H5N1 was reported in Hong Kong in 1997 with eighteen cases and six deaths [8]–[10] and the virus has continued to be associated with a high case–fatality rate [11]. Up until now, human cases have only been caused by close contact with animals (mainly poultry) infected with the virus. Although there have been isolated reports of transmission from one human to another [12], [13] the H5N1 virus does not currently fulfil the third pandemic pre-requisite which is sustained human-to-human transmission. Nevertheless the endemicity of H5N1 in poultry in many areas and the expansion of its avian and mammalian host range are providing more opportunities for human exposure [1]. This in turn increases the risk of reassortment or direct mutation into a virus better adapted for human transmission.

In the event of a pandemic, vaccination is universally regarded as the most important public health intervention for preventing influenza and reducing its health consequences [14]–[16]. The use of reverse genetics to remove the H5 polybasic amino acid sequence associated with pathogenicity has enabled production of prototype reassortant H5N1 vaccine strains containing H5 and N1 gene segments inserted into a backbone containing the other six influenza genes from PR8, a laboratory adapted avirulent H1N1 strain [17], [18].

Several H5N1 vaccines have been developed [19]–[23] and two vaccines (one split-virion [19] and one whole-virion [22]) have already been licensed [24], [25]. Indeed, many countries are now planning to amass a stockpile of H5N1 vaccine. However H5N1 vaccine stockpiles will be severely constrained by the lack of sufficient H5 vaccine antigen due to limited global production capacity. High priority has thus been given to the investigation of strategies that economize on the use of antigen such as improving immunogenicity by adjuvantation [14]. Our group recently reported on the safety and immunogenicity of an adjuvanted inactivated split-virion A/Vietnam/1194/2004 (clade 1) H5N1 candidate vaccine [23]. This study was the first to show a significant antigen dose-sparing effect induced by the inclusion of a novel adjuvant [23]. Two adjuvanted doses containing only 3.8 µg HA were found to be sufficiently immunogenic to comply with licensure criteria set out by the CHMP [15] and FDA [16]. This HA dose is more than 20 fold less than the 90 µg dose required for the H5N1 inactivated split-virion vaccine approved by the FDA [19], [25].

Another significant finding in this study was that the adjuvant also enhanced cross-reactive immunity of the A/Vietnam/1194/2004 vaccine against a prototype strain derived from the more recent H5N1 drift strain A/Indonesia/5/2005. Phylogenetic and antigenic analyses of the HA of H5N1 viruses collected since 1997 indicate that they have evolved into different sublineages or clades [18]. As we cannot predict the evolution of the H5 HA or which strain will become pandemic it will not be possible to develop a vaccine matching the actual pandemic strain for several months after its emergence. This means that advance stockpiling of vaccine is only useful if the vaccine can elicit a broad cross-protective immunity against different H5N1 viruses, including newly emerged strains. Analysis of the HA sequences of H5N1 isolates collected between August 2006 and March 2007 indicate that the majority belong to clades 1 and 2 [18]. Clade 1 viruses were found in Cambodia, Thailand and Vietnam while clade 2 viruses circulated in China and Indonesia later spreading to the Middle East, Europe and Africa. Furthermore, multiple subclades of clade 2 have been distinguished, three of which (clades 2.1, 2.2 and 2.3) have so far been largely responsible for human cases [18]. The recombinant A/Indonesia/5/2005 H5N1 strain used to assess cross-immunity in our previous report [23] belongs to clade 2.1.

Here we report on further analysis of the cross-protective potential of the candidate adjuvanted clade 1 A/Vietnam/1194/2004 vaccine and demonstrate induction of cross-clade immunity against reassortant H5N1 strains derived from clade 2.2 (A/turkey/Turkey/1/2005) and clade 2.3 (A/Anhui/1/2005) viruses as currently recommended by WHO [18]. Furthermore, we demonstrate the persistence of cross-immunity against all three clade 2 subclades at six months following vaccination.

## Methods

The CONSORT checklist and flowchart are available as supporting information; please see Checklist S1 and Flowchart S1.

We conducted a randomised, observer-blind clinical trial to assess the safety and immunogenicity of an inactivated split A/Vietnam/1194/2004 NIBRG-14 (recombinant H5N1 engineered by reverse genetics obtained from the National Institute for Biological Standards and Control (NIBSC), Potters Bar, UK) vaccine (manufactured by GlaxoSmithKline (GSK) Biologicals, Dresden, Germany). Two doses of the vaccine were administered 21 days apart to eight groups of 50 healthy male and female volunteers aged 18–60 years. Four HA antigen doses (3.8 µg, 7.5 µg, 15 µg or 30 µg) were given with or without an oil-in-water emulsion based adjuvant system [23]. The study was conducted at the Centre for Vaccinology, Ghent University and Hospital, Ghent, Belgium and is registered with the ClinicalTrials.gov registry (number NCT00309634). Written informed consent was obtained from all participants.

A detailed account of the study methodology has been published along with the results for the co-primary objectives (safety and humoral immune response) including data on cross-reactive immunogenicity induced by the low antigen dose formulations (containing 3.8 µg and 7.5 µg HA) against a heterologous strain derived by reverse genetics from the drifted clade 2 H5N1 virus, A/Indonesia/5/2005 (subclade 2.1) [23]. This present report evaluates the cross-reactive immunogenicity induced by the adjuvanted and non-adjuvanted 3.8 µg HA formulations against further heterologous strains derived by reverse genetics from drifted clade 2 H5N1 viruses A/turkey/Turkey/1/2005 (subclade 2.2, provided by NIBSC, Potters Bar, UK) and A/Anhui/1/2005 (subclade 2.3, provided by the Centers for Disease Control and Prevention, Atlanta, USA). Data are also presented on the persistence of cross-reactive antibodies against all three heterologous clade 2 H5N1 strains, which are currently recommended for use in vaccine development by WHO [18]. Cross-reactive immunogenicity was assessed by neutralisation and haemagglutination-inhibition (HAI) assays (performed as described previously [23]) on serum samples obtained at 21 days following the first vaccine dose (day 21), at 21 days following the second vaccine dose (day 42) and at approximately 6 months following vaccination (day 180).

We summarised cross-reactive immunogenicity in a subset of subjects from the per protocol population. The endpoints were neutralising seroconversion rate (at least a four-fold increase in titre relative to the pre-vaccination titre), the percentage of subjects with post-vaccination HAI titre ≥1∶40 (deemed to be the seroprotective threshold for seasonal influenza vaccines) and geometric mean titres (GMTs) at each time point (with 95% CI).

### Role of the funding source

GSK Biologicals was the funding source and was involved in all stages of the study conduct and analysis. GSK Biologicals also took in charge all cost associated to the development and the publishing of the present manuscript. The corresponding author had full access to the data and had final responsibility to submit for publication.

## Results

Four hundred adults were enrolled into the study and randomised to the eight vaccine groups, all received the two planned vaccinations and completed the study [23]. In the two study groups (non-adjuvanted 3.8 µg HA dose and adjuvanted 3.8 µg HA dose) which this report focuses on, all but one subject (who did not comply with the blood sampling schedule) were included in the per protocol cohort giving 50 subjects per group. Of these, a subset of 20 subjects from each group, selected only on the basis of the availability of a sufficient volume of serum for testing were analysed for immune responses against the recombinant A/turkey/Turkey/1/2005 and A/Anhui/1/2005 clade 2 strains. It should be noted that 43 subjects from the non-adjuvanted 3.8 µg HA group and 48 from the adjuvanted 3.8 µg HA group had previously been analysed for immune responses against the recombinant A/Indonesia/5/2005 clade 2 strain [23]. In this report we again present the A/Indonesia/5/2005 neutralising data, but only for the 20 subjects also analysed against the other clade 2 strains. The median ages (27 and 28 years) and gender ratios (75% and 80% female) of the subset populations in each group were similar. All subjects were white Caucasian.

**Figure 1:**
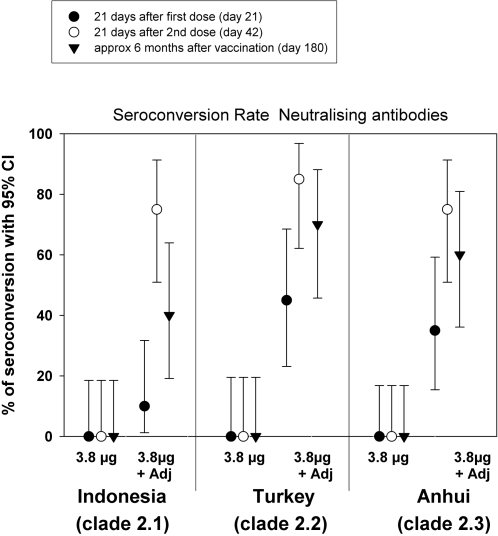
Neutralising seroconversion rates to the heterologous recombinant A/Indonesia/5/2005, A/Anhui/1/2005 and A/turkey/Turkey/1/2005 strains following vaccination with A/Vietnam/1194/2004 NIBRG-14 vaccine.

**Figure 2:**
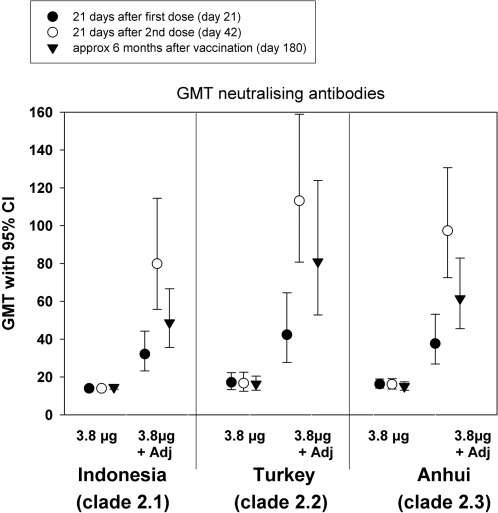
Neutralising geometric mean titres (GMTs) to the heterologous recombinant A/Indonesia/5/2005, A/Anhui/1/2005 and A/turkey/Turkey/1/2005 strains following vaccination with A/Vietnam/1194/2004 NIBRG-14 vaccine.

Figure 1 (seroconversion rates) and Figure 2 (GMTs) present the cross-neutralising responses after the first (day 21) and second (day 42) vaccine doses and at 6 months following vaccination (day 180). Four-fold seroconversion responses were recorded against all three recombinant clade 2 strains in the adjuvanted group while there was no response against any of the three strains in the non-adjuvanted group. Following the first dose, the seroconversion rates were similar for the recombinant subclade 2.2 A/turkey/Turkey/1/2005 (45%) and subclade 2.3 A/Anhui/1/2005 (35%) strains. A 10% seroconversion rate was recorded in the same subjects for the recombinant subclade 2.1 A/Indonesia/5/2005 strain. Following the second dose, seroconversion rates were similar (75%–85%) for all three strains. In most of these subjects (60–70%) the cross-neutralising response against the recombinant subclade 2.2 and 2.3 strains was still evident at 6 months following vaccination, while at this time-point 40% of subjects retained vaccine induced neutralising antibodies against the recombinant subclade 2.1 strain.

The enhancing effect of the adjuvant on the cross-neutralising responses is reflected in the GMTs against all three strains which in the non-adjuvanted group remain at pre-vaccination levels (below 20) at all time-points but in the adjuvanted group increase to between 32 to 42 after the first dose and 80 to 113 after the second dose. Again the persistence of cross-neutralising antibodies against all three strains is evident from the GMTs at 6 months following vaccination.

The HAI response (data not shown) was weaker than the neutralising response with seroprotective HAI titres induced in 35% and 60% of subjects respectively for the A/Anhui/1/2005 and A/turkey/Turkey/1/2005 strains following the second dose. Only one subject in the non-adjuvanted group had a seroprotective HAI titre against the A/turkey/Turkey/1/2005 strain.

## Discussion

We have shown that the candidate clade 1 H5N1 inactivated split-virion vaccine adjuvanted with an oil-in-water based emulsion adjuvant system can induce neutralising antibodies against recombinant strains derived from three recently emergent clade 2 viruses belonging to different subclades. Furthermore, this cross-clade immunity was induced at a low HA dose of 3.8 µg. These results are encouraging, as they demonstrate that a vaccine based on an existing H5N1 strain could potentially protect against a range of different emerging H5N1 strains. This is the concept on which the pre-pandemic immunisation strategy is based.

To be optimal for use in a pre-pandemic immunisation strategy, we need vaccines that i) are safe, ii) are highly immunogenic, iii) exhibit broad cross-immunity and iv) have long- lived immunity. As with other split-virion or whole-virion H5N1 vaccines based on strains derived by reverse genetics [19]–[22], the candidate adjuvanted vaccine derived from the 2004 H5N1 isolate A/Vietnam/1194/2004 seems to be well tolerated with an acceptable safety profile [23]. The adjuvanted vaccine was also shown to be highly immunogenic, a dose of 3.8 µg HA was sufficient to achieve immunity against the vaccine strain at a level that was acceptable for licensing in US and Europe [23]. An inactivated split-virion H5N1 vaccine has been licensed by the FDA, however this vaccine, which is administered without adjuvant is poorly immunogenic [19], [25]. Two 90 µg doses are needed to achieve the level of immunity required for licensing compared to one dose of 15 µg for conventional seasonal split-virion vaccines. Adjuvantation with aluminium was shown to only modestly improve the immunogenicity of inactivated split-virion H5N1 vaccine [20] although more promising results were achieved with whole-virion H5N1 vaccines administered with aluminium [21], [22].

As previously reported [23], after 2 administrations of 3.8 µg HA of the AS adjuvanted rH5N1 vaccine, 84% of the 50 volunteers presented seroprotective HAI titres against A/Vietnam/1194/2004 vaccine strain and 86% presented a four-fold seroconversion rate for neutralising antibodies while in the group of volunteers administered with the non-adjuvanted vaccine these percentages were 4% and 22%, respectively [23].

In this report we now provide evidence that the adjuvanted clade 1 candidate vaccine exhibits a broad cross-immunity against circulating strains shown to be responsible for human cases [18]. The effect of the oil-in-water emulsion based adjuvant system in promoting this cross-immunity was contrasting with the absence of a response in the non-adjuvanted group. We demonstrated that, in addition to the recombinant subclade 2.1 A/Indonesia/5/2005 strain, the vaccine also induced neutralising antibodies against two other recombinant strains derived from the recent drift H5N1 strains A/turkey/Turkey/1/2005 and A/Anhui/1/2005 which are, as defined by WHO, representatives of subclades 2.2 and 2.3 respectively. The ability of the vaccine to induce immunity against these three phylogenetic subclades is of relevance as, together with clade 1, they account for the majority of recent circulating H5N1 isolates and also human H5N1 cases [18].

Following the first dose of the vaccine a neutralising response against the subclades 2.2 and 2.3 was evidenced in 35%-45% of subjects. It has been estimated that a pandemic vaccine that provides even partial cross-protection (about 30%) could have substantial impact on attack rates [26], [27]. Thus in a critical situation where there is not sufficient time or supply of vaccine to administer a second dose, even one dose of the vaccine may help to reduce transmission of the pandemic virus. A high level of cross-immunity (75%–85%) against all three subclades was evident following the second dose. Furthermore we provide evidence that this cross-clade immunity is long-lived as it could still be detected in the majority of subjects at six months following vaccination. The neutralising antibody titres against A/Vietnam/1194/04 homologous virus follow the same trend as the cross-reactive antibody titres against clade 2 viruses (data not shown, manuscript under preparation). Humoral immunity for influenza vaccines has conventionally been assessed by HAI. Our previous experience with A/Indonesia/5/2005 H5N1 strain has shown that cross-reactivity is stronger when assessed by the more sensitive neutralisation assay [23] which provides an evaluation of the vaccine activity against both the HA and the NA antigens and consequently, gives a more comprehensive evaluation of the biological activity of the vaccine. This was confirmed in this present study for the recombinant A/turkey/Turkey/1/2005 and A/Anhui/1/2005 strains.

A recent pre-clinical study provides further evidence that vaccination with H5 and N1 antigens from one clade can induce a broadly protective immune response against wild type viruses from another clade [28]. Suguitan and colleagues showed that vaccines developed from attenuated strains containing H5 and N1 components from 1997 clade 3, 2003 clade 1 or 2004 clade 1 isolates protected mice from lethal challenge with both homologous and heterologous wild type viruses including more recent 2005 clade 1 and clade 2 viruses [28]. Similar data were generated for protection against pulmonary replication following challenge with these different strains in vaccinated mice and ferrets. The authors suggested that the high level of protection afforded by vaccination with the 1997 clade 3 vaccine against challenge with the clade 1 and clade 2 H5N1 viruses isolated over a span of 8 years, indicates that the H5N1 viruses are evolving to infect different birds, and not predominantly to evade antibodies as they do in humans [28]. Earlier evidence that an avian influenza vaccine could exhibit cross-immunity came from a study where a surface-antigen vaccine based on the antigenically related H5N3 influenza virus (influenza A/duck/Singapore/97) and adjuvanted with MF59 induced cross-reactive antibodies against H5N1 [29]. Whereas we did not observe any cross-reactive response following administration of two doses of non-adjuvanted vaccine, these authors did measure some degree of cross-reactivity after three doses of non-adjuvanted vaccine.

We have previously demonstrated the significant antigen dose-sparing effect of including an oil-in-water emulsion based adjuvant system in the candidate vaccine formulation [23]. This is now re-enforced by the results of this present study which confirm that the adjuvant enhances the effectiveness of a low antigen dose in broadening the immune response. Baras et al [30] recently documented in a stringent preclinical model that the AS adjuvanted candidate vaccine described in the present paper provides protection against cross-clade heterologous challenge in ferrets. The availability of H5N1 vaccines that can elicit a broad cross-protective immunity against different currently circulating H5N1 viruses, including newly emerged strains, is a pre-requisite for the development of a successful pre-pandemic vaccination strategy. Deployment of such vaccines for pre-emptive vaccination could play a key role in pandemic mitigation during the several months that it would take to produce an H5N1 vaccine exactly matched to a pandemic strain.

## Supporting Information

Checklist S1 CONSORT Checklist
(0.13 MB PDF)

Flowchart S1 CONSORT Flowchart
(0.11 MB PDF)
