# Supplementary material for: Broad Clade 2 Cross-Reactive Immunity Induced by an Adjuvanted Clade 1 rH5N1 Pandemic Influenza Vaccine
Source: PLoS One. 2008 Feb 27;3(2):e1665. doi: 10.1371/journal.pone.0001665 (PMC2253495; doi:10.1371/journal.pone.0001665)

# The Consort E-Flowchart  Aug. 2005

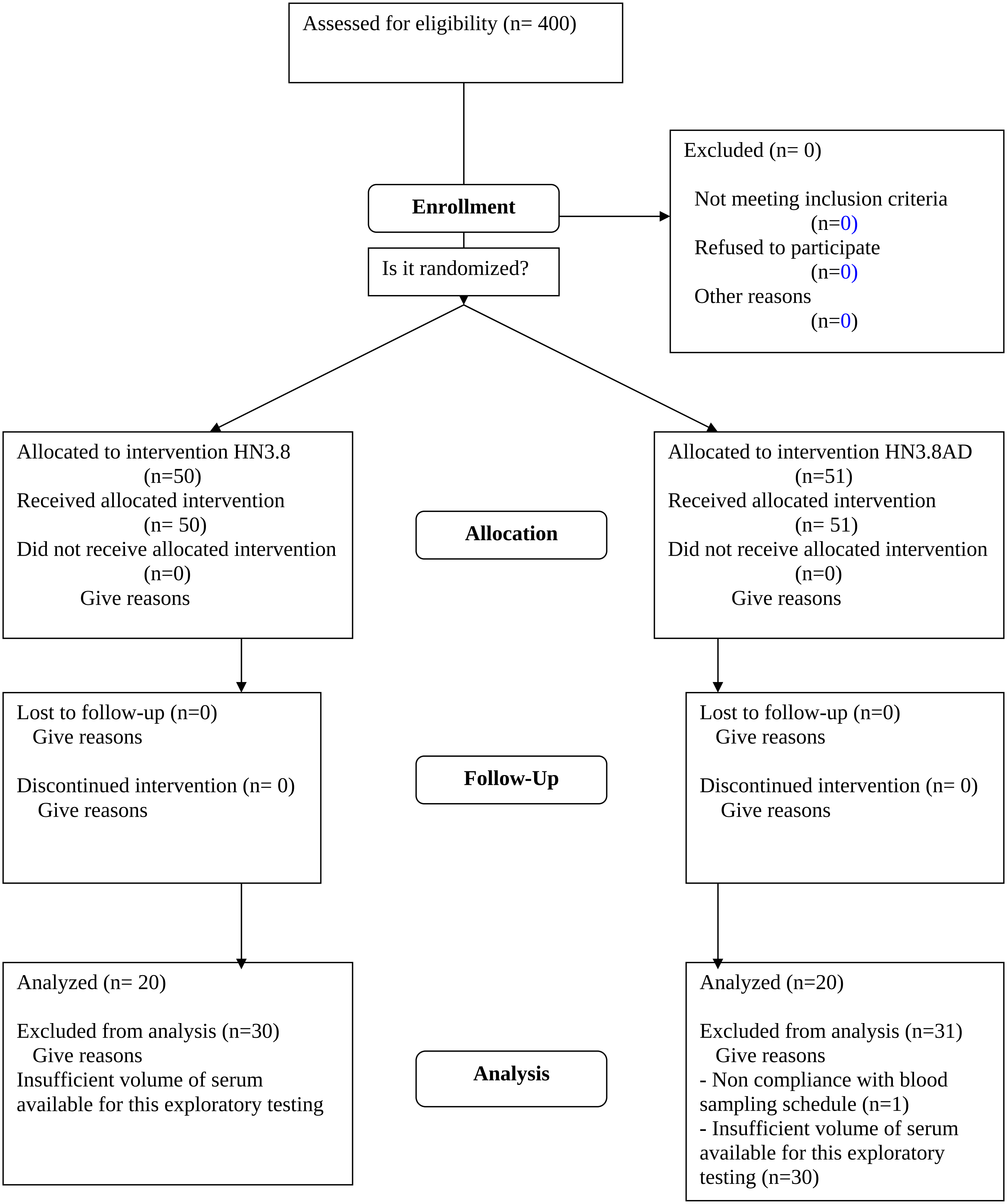

# The Consort E-Flowchart Aug. 2005

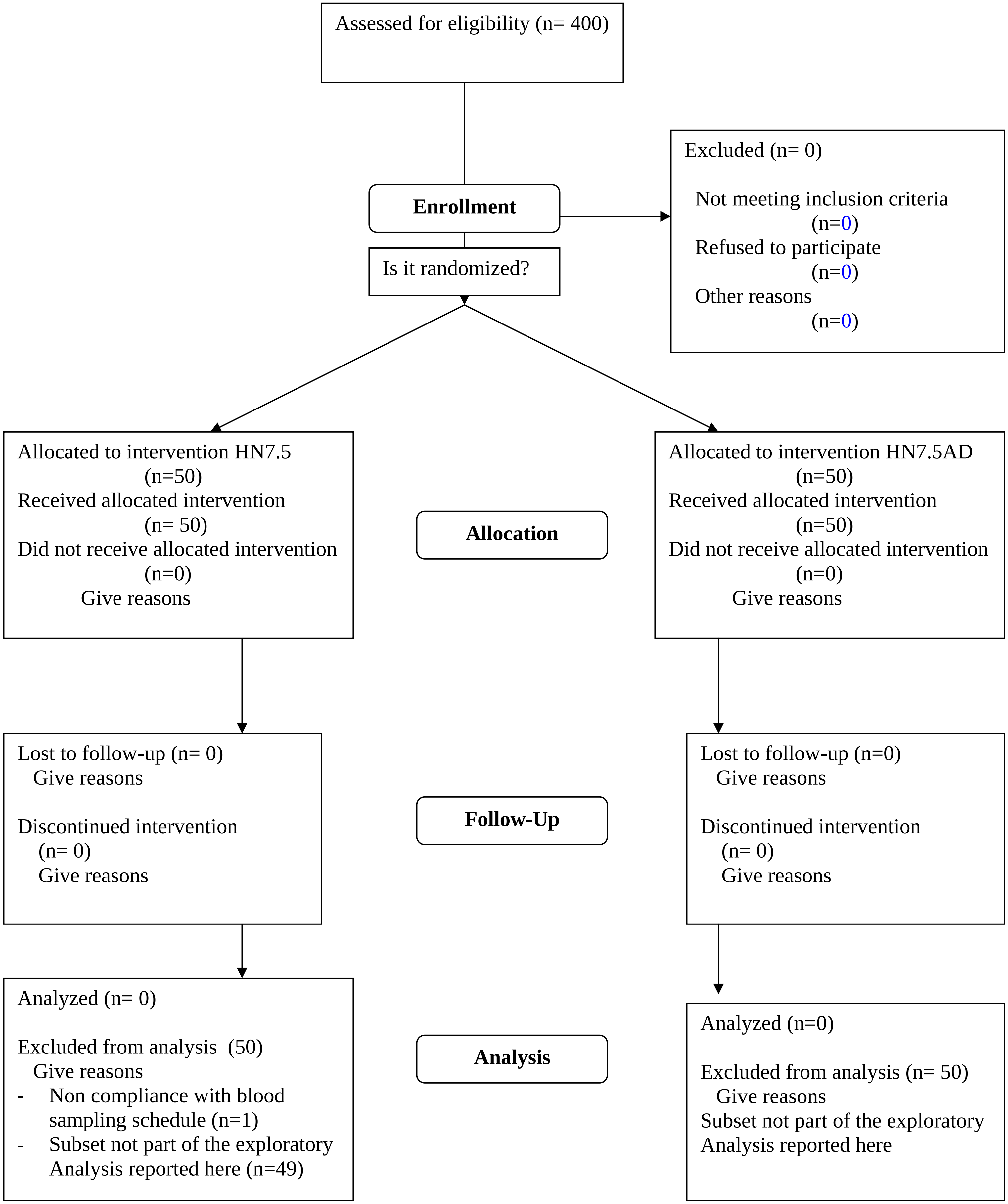

# The Consort E-Flowchart  Aug. 2005

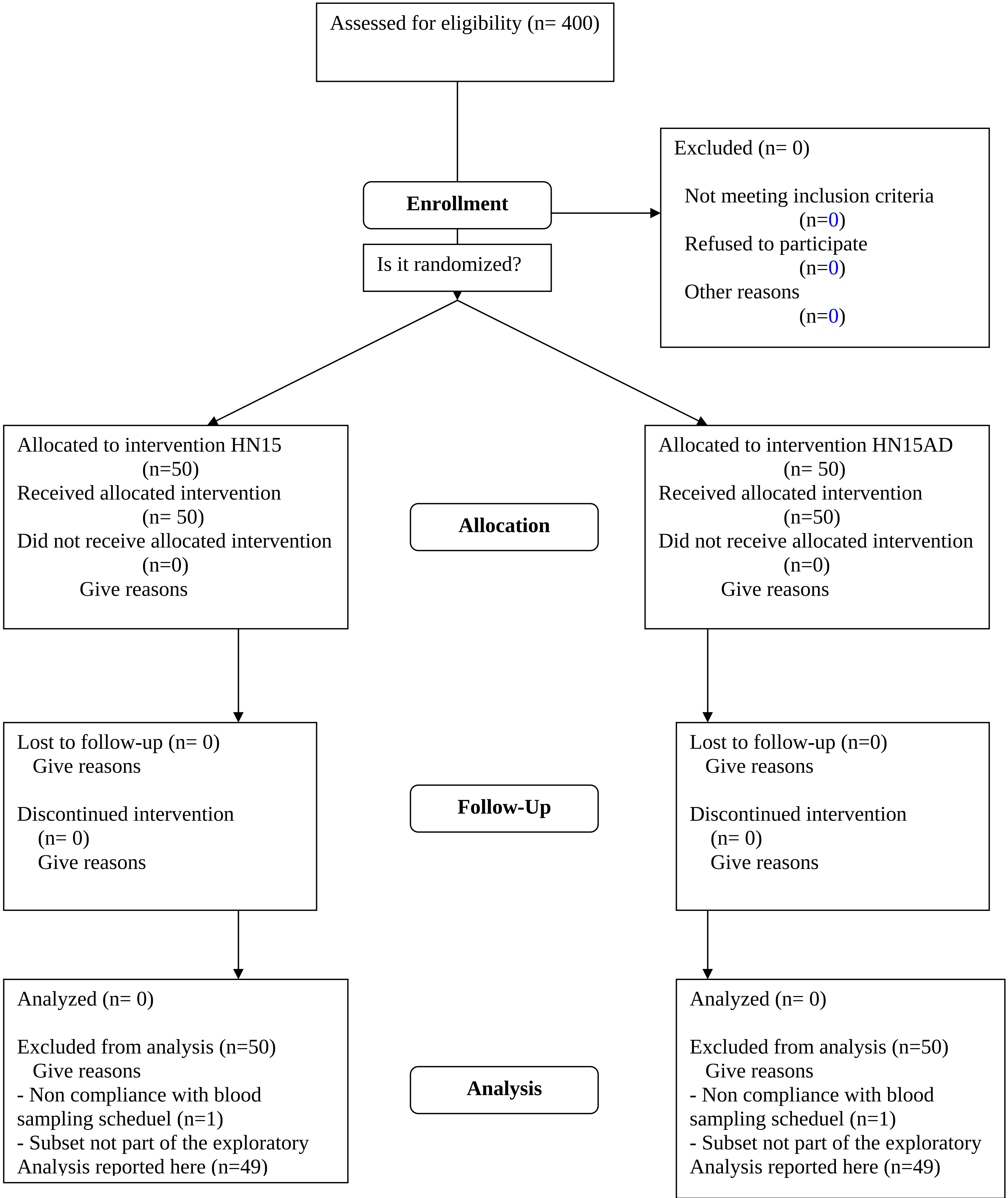

# The Consort E-Flowchart Aug. 2005

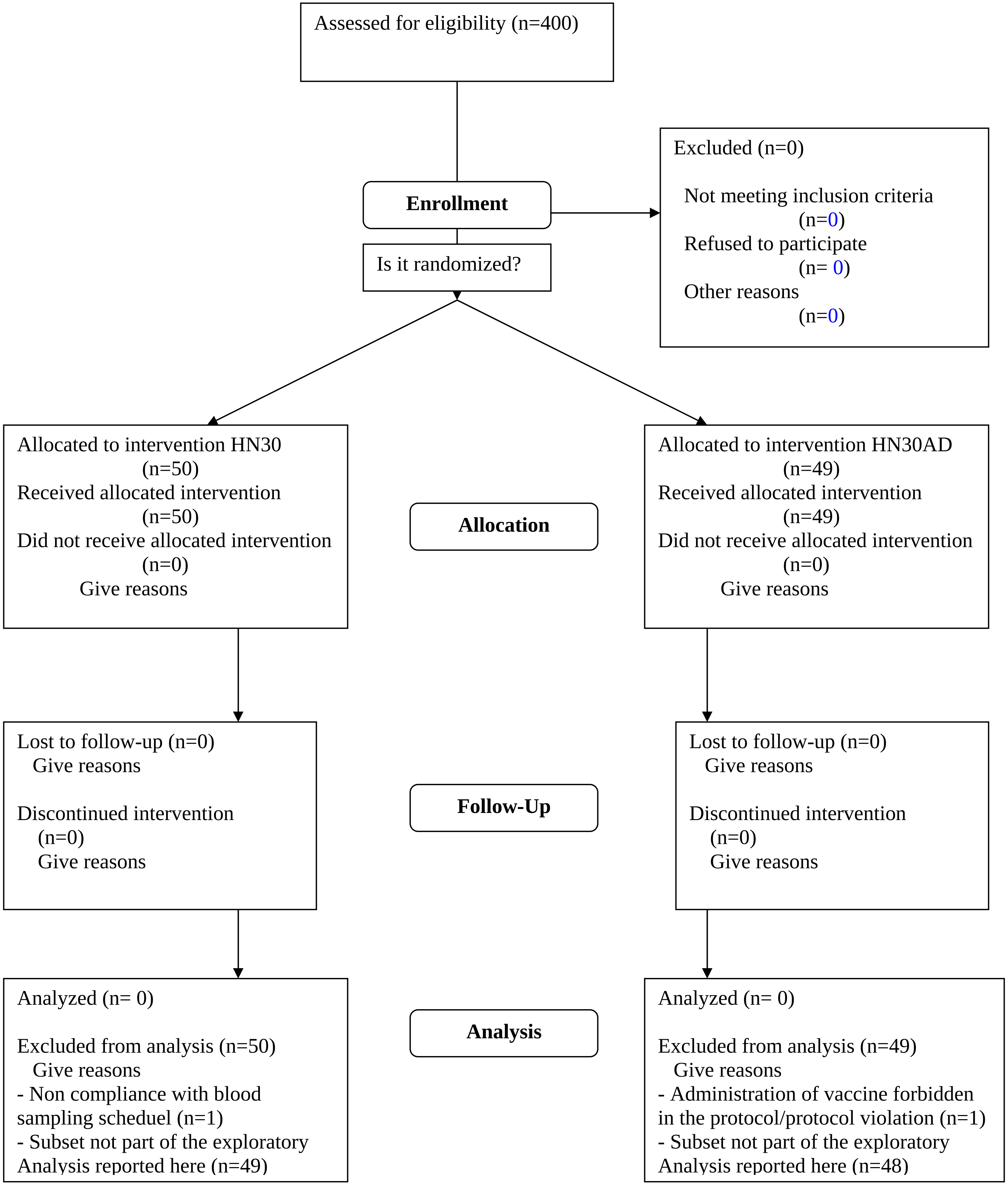

Supplement: Flowchart S1 — CONSORT Flowchart (0.11 MB PDF) [file pone.0001665.s002.pdf]
